# CD4^+^ T cell help creates memory CD8^+^ T cells with innate and help-independent recall capacities

**DOI:** 10.1038/s41467-019-13438-1

**Published:** 2019-12-04

**Authors:** Tomasz Ahrends, Julia Busselaar, Tesa M. Severson, Nikolina Bąbała, Evert de Vries, Astrid Bovens, Lodewyk Wessels, Fred van Leeuwen, Jannie Borst

**Affiliations:** 1 Division of Tumor Biology and Immunology, The Netherlands Cancer Institute-Antoni van Leeuwenhoek, 1066 CX Amsterdam, The Netherlands; 2 Division of Molecular Carcinogenesis, Oncode Institute, The Netherlands Cancer Institute-Antoni van Leeuwenhoek, 1066 CX Amsterdam, The Netherlands; 3 Division of Gene Regulation, The Netherlands Cancer Institute-Antoni van Leeuwenhoek, 1066 CX Amsterdam, The Netherlands; 4 Present Address: Laboratory of Mucosal Immunology, The Rockefeller University, New York, NY USA; 5 Present Address: Department of Immunohematology and Blood Transfusion, Leiden University Medical Center, Leiden, The Netherlands

**Keywords:** Immunological memory, Interleukins, CD4-positive T cells, Cytotoxic T cells

## Abstract

CD4^+^ T cell help is required for the generation of CD8^+^ cytotoxic T lymphocyte (CTL) memory. Here, we use genome-wide analyses to show how CD4^+^ T cell help delivered during priming promotes memory differentiation of CTLs. Help signals enhance IL-15-dependent maintenance of central memory T (T_CM_) cells. More importantly, help signals regulate the size and function of the effector memory T (T_EM_) cell pool. Helped T_EM_ cells produce Granzyme B and IFNγ upon antigen-independent, innate-like recall by IL-12 and IL-18. In addition, helped memory CTLs express the effector program characteristic of helped primary CTLs upon recall with MHC class I-restricted antigens, likely due to epigenetic imprinting and sustained mRNA expression of effector genes. Our data thus indicate that during priming, CD4^+^ T cell help optimizes CTL memory by creating T_EM_ cells with innate and help-independent antigen-specific recall capacities.

## Abstract (web summary)

Help from CD4^+^ T cells is important to induce CD8^+^ T cell memory responses, but mechanistic insights are lacking. Here, the authors show, by transcriptomics and epigenetics, how CD4^+^ T cells help program memory CD8^+^ T cells for help-independent recall by antigens, as well as for innate-like recall responses by IL-12/IL-18 and promoting survival by IL-15.

## Introduction

Immunological memory is classically attributed to cells of the adaptive immune system. It is based on clonal expansion of antigen-specific lymphocytes, long-term maintenance of at least part of this antigen-specific lymphocyte pool as memory cells and the intrinsic ability of memory cells to respond more efficiently to renewed antigen encounter^1^. Memory CD8^+^ T cells can also be reactivated by cytokines in an antigen-independent manner^2^.

A single activated CD8^+^ cytotoxic T lymphocyte (CTL) can give rise to both effector and memory cells^3^. The differentiation path of a given CD8^+^ T cell is graded from less to more differentiated, culminating in the terminally differentiated effector cell that is short-lived. Compared to effector cells, memory CD8^+^ T cells reportedly exhibit less differentiated phenotypes, can self-renew, have high proliferative potential and increased longevity^4^. Memory CD8^+^ T cells are discerned into central (T_CM_), effector (T_EM_) and tissue-resident (T_RM_) cells^5^. T_CM_ cells and T_EM_ cells can be discriminated based on expression of homing receptors that allow T_CM_ cells to circulate through lymph nodes, while T_EM_ cells primarily circulate through non-lymphoid tissues and spleen.

Whether a primed CD8^+^ T cell becomes a short-lived effector cell (SLEC), or a memory precursor cell (MPEC) is decided upon by specific transcription factors that act in pairs to determine opposite cell fates by directing specific gene expression programs^3,6^. For example, the balance between Blimp-1 and Bcl-6 is decisive for an effector versus memory T cell fate. Cell fate decisions are largely based on gene transcription that is orchestrated at the epigenetic level^7^. Epigenetic processes, such as DNA methylation, histone acetylation, and histone methylation confer chromatin states of a gene that facilitate or prohibit transcription. Recent data reveal a coupling between the master regulators of transcription and epigenetic modification^6,8^.

Memory CD8^+^ T cells have an intrinsic ability to more efficiently expand and display effector functions upon secondary stimulation. Certain genes are more readily expressed in activated memory T cells as compared to activated naïve T cells^7,9^. This may be due to the fact that these genes are epigenetically poised, which means that they have an open chromatin state, but low gene expression in the steady-state memory phase^9^. For example, the promotor regions of the *Il2* and *Ifng* genes are more rapidly demethylated upon recall^10,11^. Alternatively, genes can already be expressed in steady-state memory cells at the mRNA level, but not at the protein level. Recall with antigen, but also with cytokines can induce protein translation from such transcripts and thereby efficient recall of functions^12^.

Intrinsic memory qualities are instilled into CD8^+^ T cells during the priming phase, a phenomenon termed memory programming^13^. The generation and programming of memory CD8^+^ T cells relies on “help” signals that are delivered by CD4^+^ T cells during priming^14–17^. These help signals are relayed from the CD4^+^ T cell to the CD8^+^ T cell via an XCR1^+^ lymph-node resident dendritic cell (DC), as established in the mouse^17,18^. The DC is conditioned by the CD4^+^ T cell to deliver certain costimulatory signals and cytokines that orchestrate CTL effector- and memory differentiation^14,15,17,19^. We have recently identified by transcriptomic and functional analyses the gene expression program underlying CTL effector differentiation as instructed by CD4^+^ T cell help^20^.

We here present the impact of CD4^+^ T cell help on the gene expression program of steady-state memory CD8^+^ T cells and secondary effector CTLs. We demonstrate that help delivered during priming promotes the size of both T_CM_ and T_EM_ pools, but primarily alters the intrinsic functionality of T_EM_ cells. Remarkably, help signals confer cytokine-induced and help-independent recall capacities to CD8^+^ memory T cells and allow them to “remember” that they have received help during priming.

## Results

### Help endows CD8^+^ T cells with intrinsic memory capacity

**Fig. 1:**
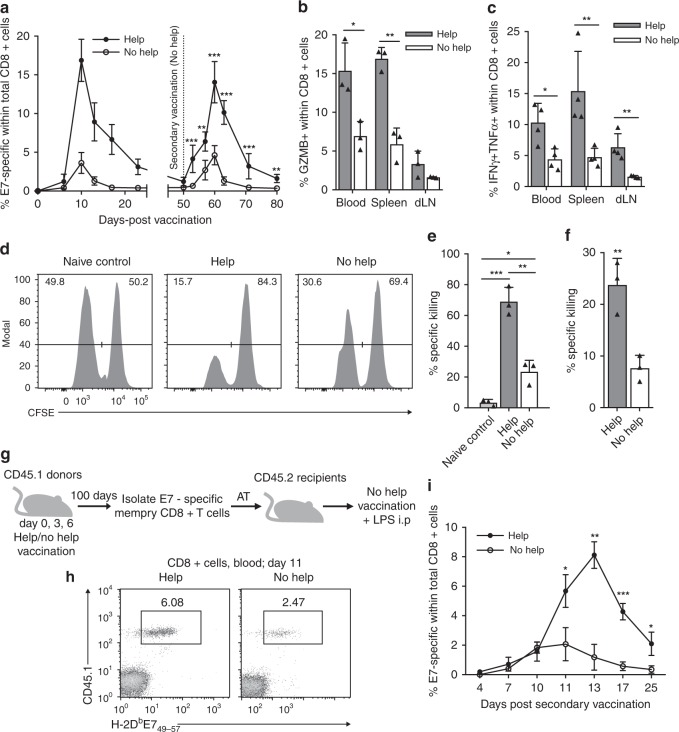
CD4^+^ T cell help instills intrinsic recall capacities into CD8^+^ T cells. **a**–**f** Mice were vaccinated intra-epidermally with a DNA construct encoding HPV-E7 with (Help) or without (No Help) MHC class II-restricted epitopes on days 0, 3, and 6. On day 50, mice were rechallenged with No Help vaccine and i.p. lipopolysaccharide (LPS) injection. (A) Percentage of H-2D^b^/E7_49-57_ tetramer^+^ cells among total CD8^+^ T cells in blood at indicated days after first vaccination (*n* = 7 per group). **b**, **c** In a separate experiment, on day 10 after rechallenge, frequencies of Granzyme B (GZMB)^+^ cells (B) and IFNγ^+^TNFα^+^ cells (C) within total CD8^+^ T cells in indicated tissues were determined by flow cytometry (*n* = 3–4). **d**, **e** In a separate experiment, mice (*n* = 3) were injected i.v on day 10 after rechallenge with a 1:1 ratio of splenocytes loaded with E7_49-57_ peptide (CTL target) or not (control) and labeled with a low or high dose of carboxyfluorescein succinimidyl ester (CFSE), respectively. Naive recipient mice were used as control. After 15 h, percentage of specific in vivo target cell killing was determined by flow cytometric analysis of splenocytes. **d** Representative histograms of CFSE^+^ cells. **e** Bar diagram quantitatively depicting all results. **f** In a separate experiment, E7-specific CD8^+^ T cells were isolated from spleen at day 10 after rechallenge (*n* = 3). T cells were incubated in vitro at a 1:1 ratio with E7_49-57_-peptide-loaded or non-loaded splenocytes and specific killing of target cells was analyzed 12 h later. **g**–**i** CD45.1^+^ donor mice received Help or No Help vaccine. At day 100, E7-specific (memory) CD8^+^ T cells were isolated from spleens and injected i.v. in equal numbers into CD45.2^+^ recipient mice (*n* = 4 per group). One day later, recipient mice were rechallenged. **g** Experimental set up. **h** Representative flow cytometry plots indicating frequencies of CD45.1^+^ E7-specific CD8^+^ T cells in blood at day 11 after rechallenge. **i** Frequencies of CD45.1^+^ E7-specific CD8^+^ T cells in blood at indicated days after vaccination. Data in this figure are from one experiment representative of at least two experiments. Error bars indicate SD, *p < 0.05, **p < 0.01, ***p < 0.001 (unpaired two-tailed Student’s *t* test). Source data are provided as a Source Data file.

To determine how CD4^+^ T cell help delivered during priming impacts CD8^+^ T cell memory, we used a mouse model of therapeutic vaccination. A comparative setting was created using two plasmid (p)DNA vaccines that encode the human papilloma virus (HPV) E7 protein either with the immunodominant, MHC class I-restricted epitope E7_48-57_ alone (No Help), or in conjunction with exogenous, HPV-unrelated MHC class II-restricted helper epitopes (Help)^21^. As shown before^20–22^, inclusion of helper epitopes in the vaccine significantly increased the magnitude of the primary H-2D^b^/E7_48-57_ (E7)-specific CD8^+^ T cell response (Fig. 1a, Supplementary Fig. 1A, B). Help also significantly increased the total numbers of E7-specific CD8^+^ T cells with a SLEC phenotype (CD127^-^KLRG1^+^), as well as those with MPEC phenotype (CD127^+^KLRG1^−^) (Supplementary Fig. 1C).

To examine the impact of help delivered during priming on the memory CD8^+^ T cell response, mice were primed with either Help or No Help vaccine and recalled with No Help vaccine in conjunction with i.p. injection of lipopolysaccharide (LPS)^22^. Mice primed with the Help vaccine had a significantly higher recall response to H-2D^b^/E7_48-57_ than mice primed with No Help vaccine (Fig. 1a). At the peak of the secondary response, the frequencies of CD8^+^ T cells expressing Granzyme B (Fig. 1b), IFNγ and TNFα (Fig. 1c) in blood, draining lymph node (dLN) and spleen were significantly higher after priming with Help as compared to No Help vaccine. Accordingly, an in vivo cytotoxicity assay revealed that—at the peak of the secondary response—injected E7_49-57_ peptide-loaded target cells were killed much more efficiently in mice primed with the Help vaccine, than in mice primed with the No Help vaccine (Fig. 1d, e). Thus, CD4^+^ T cell help delivered during priming improved the secondary CD8^+^ T cell response to MHC class I-restricted antigen only.

The secondary CD8^+^ T cell response might be improved because help signals lead to increased numbers of E7_48-57_-specific memory CD8^+^ T cells and/or improve their cell-intrinsic capacity to undergo a secondary response. In vitro, secondary responder CTLs that had received help during priming could kill target cells more efficiently than equal numbers of helpless secondary responder CTLs (Fig. 1f), arguing that help signals improved memory CTL function on a per-cell basis. To examine this further, we performed an adoptive transfer experiment. Memory CD8^+^ T cells isolated from mice primed either with Help or No Help vaccine were transferred in equal numbers to naïve recipient mice that were subsequently challenged with No Help vaccine (Fig. 1g). Memory CD8^+^ T cells isolated from mice primed with Help vaccine expanded significantly more than those primed with No Help vaccine (Fig. 1h, i). Thus, CD4^+^ T cell help delivered during priming instills into memory CD8^+^ T cells cell-intrinsic capacities for CD4^+^ T cell help-independent secondary expansion and display of cytotoxic effector function.

### Help supports CD8^+^ T_EM_ and T_CM_ formation and maintenance

**Fig. 2:**
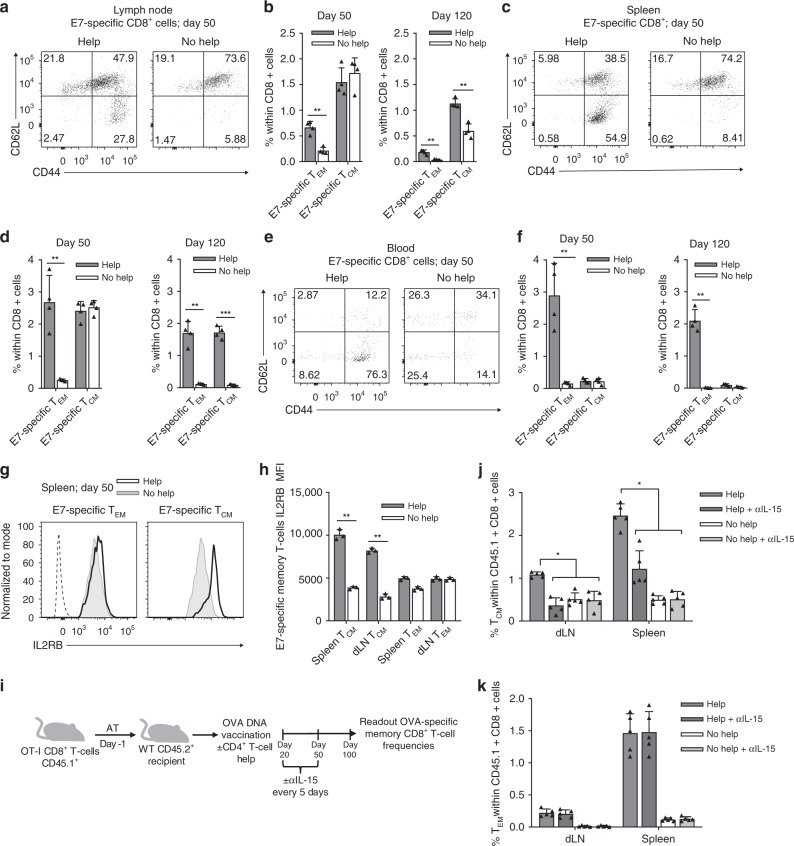
CD4^+^ T cell help supports formation of CD8^+^ T_CM_ and T_EM_ cells. **a**–**f** Mice (*n* = 4 per group) received Help or No Help vaccine on days 0, 3, and 6 and were analyzed on day 50 or 120. **a**, **c**, **e** Representative flow cytometric plots indicating within the H-2D^b^/E7_49-57_ tetramer^+^ CD8^+^ population frequencies of cells with an effector memory T (T_EM_) phenotype (CD44^+^CD62L^−^) or central memory T (T_CM_) phenotype (CD44^+^CD62L^+^), as determined in draining lymph node (dLN) (**a**), spleen (**c**), and blood (**e**). **b**, **d**, **f** Quantification of frequencies of E7-specific CD44^+^CD62^-^ T_EM_ and CD44^+^CD62^+^ T_CM_ phenotype CD8^+^ T cells determined in dLN (**b**), spleen (**d**), and blood (**f**). **g**, **h** Cell surface expression of IL2RB (CD122) as determined by flow cytometry on E7-specific CD8^+^ T_CM_ and T_EM_ cell subsets in spleen at day 50. **g** Primary data. Filled grey: No Help; open black line: Help. Dotted line: unstained control. **h** Quantification of mean fluorescence intensity (MFI) for *n* = 3. **i** Experimental set up. CD45.1^+^ OT-I T cells were adoptively transferred into CD45.2^+^ recipient mice (*n* = 5 per group) that 1 day later received OVA-encoding Help or No Help vaccine. From day 20 onwards, indicated groups were treated with isotype control antibody or neutralizing αIL-15 antibody every 5 days until day 50. **j**, **k** Frequencies of CD45.1^+^ T_CM_ cells (**j**) and T_EM_ cells (**k**) were determined at day 100 in dLN and spleen. Data in this figure are from one experiment representative of two experiments. Error bars indicate SD, *p < 0.05, **p < 0.01, ***p < 0.001 (unpaired two-tailed Student’s *t* test). Source data are provided as a Source Data file.

We next tested in the same vaccination settings how CD4^+^ T cell help impacted the establishment and maintenance of CD8^+^ T_EM_ and T_CM_ subpopulations in secondary lymphoid tissues and blood. The frequencies of E7-specific CD8^+^ T cells with a T_EM_ phenotype (CD44^+^CD62L^−^) and a T_CM_ phenotype (CD44^+^CD62L^+^) were determined by flow cytometry. At day 50, the frequency of E7-specific T_EM_ cells among total CD8^+^ T cells in the dLN was significantly lower after No Help vaccination than after Help vaccination (Fig. 2a, b). At the same time point, there were no significant differences in the frequencies of E7-specific T_CM_ cells in the two vaccination settings (Fig. 2a, b). At day 120, however, the frequencies of both E7-specific T_EM_ cells and T_CM_ cells were significantly lower after No Help vaccination than after Help vaccination (Fig. 2b). Likewise in the spleen, only the T_EM_ cell pool was significantly smaller after No Help vaccination than after Help vaccination at day 50 (Fig. 2c, d), while at day 120, both T_EM_ and T_CM_ pools were significantly smaller after No Help vaccination than after Help vaccination (Fig. 2d).

T_EM_ cells circulate through the blood and secondary lymphoid organs^4,5^ and accordingly, the beneficial influence of help on the size of the T_EM_ cell pool in blood was evident both at day 50 and day 120 (Fig. 2e, f). At both time points, frequencies of T_CM_ cells in blood were very low, in agreement with their preferential localization to secondary lymphoid organs^4,5^ (Fig. 2e, f). In the bone marrow, T_EM_ frequencies were reduced in the helpless population as compared to the helped population on day 50 and this difference was more significant on day 120 (Supplementary Fig. 2A, B). We next examined the effect of CD4^+^ T cell help on the generation of memory CD8^+^ T cell subsets in a model of overt inflammation. Mice were infected with the acute lymphocytic choriomeningitis virus (LCMV) strain Armstrong and depleted for CD4^+^ T cells (No Help) or not (Help). At day 50 after infection we analyzed frequencies of GP33- and NP396-specific T_EM_ and T_CM_ cells in the spleen. Similarly to the vaccination model, help resulted in increased frequencies of both virus-specific memory subsets (Supplementary Fig. 2C). Thus, CD4^+^ T cell help delivered during priming improved generation of the CD8^+^ T_EM_ population and long-term maintenance of both CD8^+^ T_EM_ and T_CM_ populations.

Memory CD8^+^ T cells are known to undergo antigen-independent homeostatic proliferation driven by IL-15^23^. At day 50 after vaccination, IL2RB (CD122), the common β chain of the IL-2- and IL-15 receptors, was expressed at significantly higher levels at the cell surface of helped as compared to helpless E7-specific T_CM_ cells, in both dLN and spleen (Fig. 2g, h). In T_EM_ cells, however, “help” did not affect the cell surface expression of IL2RB (Fig. 2g, h). These data suggested that IL-15 receptor signaling was instrumental in maintaining T_CM_ cells in dLN and spleen after Help vaccination. To test this, we performed an intervention experiment with neutralizing antibody to IL-15. OVA_257-264_/H-2K^b^-specific OT-I T cells were transferred into CD45.2^+^ recipient mice that received Help or No Help vaccine encoding the OVA_257-264_ epitope. The primary response showed the impact of help on the OT-I T cell response (Supplementary Fig. 2D). Next, mice were treated from day 20 to 50 with control antibody or neutralizing antibody to IL-15 (Fig. 2i). At day 100, the size of the T_CM_ and T_EM_ cell pools in dLN and spleen was determined. IL-15 neutralization had significantly diminished the size of the T_CM_ cell pools after Help vaccination, but did not affect the T_CM_ cell pools after No Help vaccination (Fig. 2j). The T_EM_ cell populations in dLN and spleen were unaffected by IL-15 neutralization after Help or No Help vaccination (Fig. 2k). These data indicate that help signals delivered during priming lead to upregulation of IL2RB on CD8^+^ T_CM_ cells, which likely increases their capacity for IL-15-driven long-term survival in vivo. The same help signals apparently promote long-term survival of CD8^+^ T_EM_ cells by an IL-15-independent mechanism.

### Help predominantly affects the transcriptome of T_EM_ cells

**Fig. 3:**
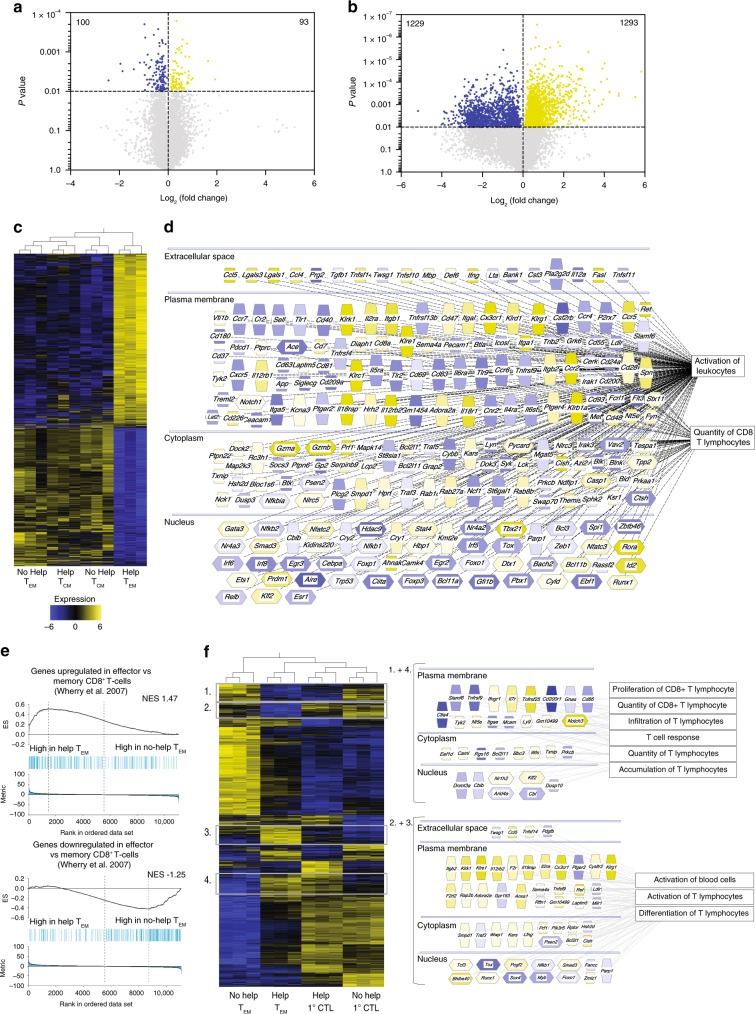
CD4^+^ T cell help primarily alters the steady-state transcriptome of T_EM_ CD8^+^ T cells. **a**, **b** Volcano plots depicting results of comparative transcriptome analysis of steady-state E7-specific CD8^+^ T_CM_ cells (**a**) and T_EM_ cells (**b**) isolated from spleen at day 100 after primary vaccination with Help or No Help vaccine (*n* = 3 mice per group). Blue and yellow dots indicate transcripts with significant differential expression (*p* < 0.01). See also Supplementary Data 1 and 2. **c** Hierarchical clustering and heat map of the differentially expressed genes in steady-state T_EM_ and T_CM_ cells taken from the Help versus No Help vaccination settings. **d** Molecules differentially expressed in steady-state T_EM_ cells after Help versus No Help vaccination, as assigned to the indicated functional categories and annotated for subcellular localization by IPA. **e** Gene Set Enrichment Analysis (GSEA) of published gene sets listing the top 200 up- or downregulated genes in effector CD8 T cells (day 8 after LCMV-Armstrong infection)^30^ within the gene expression profile of helped versus helpless T_EM_ cells in steady-state. ES, enrichment score; NES, normalized enrichment score. **f** Hierarchical clustering and heat map of genes differentially expressed in steady-state T_EM_ cells taken at day 100 after primary vaccination with Help or No Help vaccine, as determined here and genes differentially expressed in primary CTLs taken at day 10 from the same settings, as determined previously^20^. Numbers and adjacent boxes (right panels) indicate Cluster 1, denoting molecules differentially expressed in both helped versus helpless T_EM_ cells and primary CTLs and Cluster 2, denoting molecules only differentially expressed in helped versus helpless T_EM_ cells, as assigned to the indicated functional categories and annotated for subcellular localization by IPA.

To gain insight into the molecular mechanisms by which CD4^+^ T cell help altered the cell-intrinsic functionality of steady-state CD8^+^ T_EM_ and T_CM_ cells, we performed gene expression profiling. E7-specific memory CD8^+^ T_EM_ and T_CM_ cells were flow cytometrically isolated at day 100 from the spleens of mice that had received Help or No Help vaccine, mRNA was isolated and subjected to sequencing (RNAseq). This unbiased, genome-wide analysis revealed that CD4^+^ T cell help affected T_CM_ cells much less than T_EM_ cells (Fig. 3a, b). In T_CM_ cells, we found only 193 genes with significant differential expression between the comparative groups (Fig. 3a; Supplementary Data 1), but in T_EM_ cells, we found 2522 differentially expressed genes (Fig. 3b; Supplementary Data 2). Importantly, the effect size was also larger in the latter case. This is confirmed by hierarchical clustering and inspection of the resulting heatmap, revealing that T_EM_ cells dramatically change their gene expression profile as a result of CD4^+^ T cell help, while T_CM_ cells do so to a much lesser extent (Fig. 3c).

Ingenuity pathway analysis (IPA) gave insight in the many molecules that were differentially expressed in helped versus helpless T_EM_ cells (Fig. 3d). Genes with increased expression included those encoding transcription factors Tbet (*Tbx21*), Blimp-1 (*Prdm1*), Runx1 and Id2, known to be involved in CD8^+^ T cell effector function^6^. In addition, transcription factors Tcf-1 (*Tcf7*) and TOX (*Tox*), which are associated with decreased CD8^+^ T cell effector functions and an exhausted phenotype^24–26^, were specifically downregulated in T_EM_ cells as a result of CD4^+^ T cell help. Transcripts encoding the cytotoxic effector molecules Granzyme A, Granzyme B (*Gzma, Gzmb*), Perforin (*Prf1*), IFNγ, Fas ligand (*Faslg*) and Trail (*Tnfsf10*) were upregulated, indicating increased cytotoxic potential. Transcripts encoding the IL-2 receptor α chain (*ll2ra*), IL-12 receptor β chain (*Il12rb1, 2*) and IL-18 receptor α chain (*Il18r1*) were upregulated, which fits with memory T cell survival and function^2,27,28^. Several chemokine receptor transcripts were upregulated, including *Cx3cr1* that we previously found to report CD4^+^ T cell help in effector CTLs^21^. Other interesting features included the upregulation of Pycard (Asc), Caspase-1 (*Casp-1*) and NOD-like receptors (*Nlrc3, 5*) that can together create “inflammasome” structures leading to pro-IL1β processing^29^, which suggests an innate-type alarm state of helped T_EM_ cells.

These findings suggested that helped T_EM_ cells have a more effector-like phenotype than their non-helped counterparts. To examine this, we compared by Gene Set Enrichment Analysis (GSEA) the Help versus No Help T_EM_ gene expression profiles to a published gene expression profile characteristic of effector CD8^+^ T cells^30^. This gene set consisted of the top 200 up- and downregulated genes in effector (day 8) versus memory (day > 30) virus-specific CD8^+^ T cells isolated after acute LCMV-Armstrong infection. The effector CD8^+^ T cell gene set was indeed enriched in the expression profile of helped steady-state T_EM_ cells (Fig. 3e), indicating a more effector-like phenotype in this population.

To examine whether helped T_EM_ cells, isolated at the steady-state memory phase, resembled helped CTLs, isolated at the primary effector phase, we performed hierarchical clustering of all genes that were differentially expressed as a result of help in primary CTLs or in T_EM_ cells. A number of these genes was differentially expressed both in helped primary CTLs and helped T_EM_ cells (Fig. 3f, clusters 1 and 4), suggesting that part of the help signature seen in primary effectors is maintained in steady-state T_EM._ Other gene expression differences resulting from help were more pronounced in T_EM_ cells than in primary CTLs (Fig. 3f, clusters 2 and 3). This included the upregulation of transcripts for various Killer cell lectin-like receptors, the chemokine receptor CX_3_CR1, and cytokine receptors: IL2R, IL12R and IL18R (Fig. 3f). Taken together, these results show that besides increasing the size of the T_EM_ pool, CD4^+^ T cell help delivered during priming also creates a T_EM_ population with distinct, more effector-like properties.

### Help promotes innate memory function of CD8^+^ T cells

**Fig. 4:**
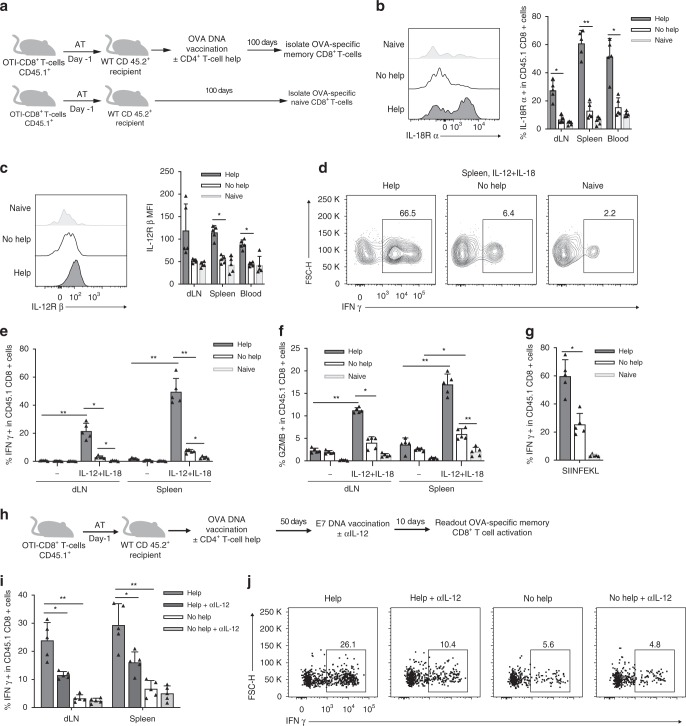
CD4^+^ T cell help promotes innate recall function of memory CD8^+^ T cells. **a** Experimental set up. CD45.1^+^ OT-I T cells were adoptively transferred into CD45.2^+^ recipient mice (*n* = 5 per group) that 1 day later received OVA-encoding Help or No Help pDNA vaccine. At day 100 after primary vaccination, flow cytometry was performed in blood and CD45.1^+^ OT-I T cells were flow cytometrically isolated from dLN and spleen and directly analyzed or incubated for 5 h with IL-12 and IL-18 or OVA peptide. Unvaccinated recipient mice were used to isolate naïve OT-I T cells. **b** Representative flow cytometric plots (left panel) and percentage of IL18-Rα expressing cells within CD45.1^+^ OT-I cells in indicated organs (right panel). **c** Representative flow cytometric plots (left panel) and MFI of IL-12Rβ expressed on CD45.1^+^ OT-I T cells in indicated organs (right panel). **d**–**g** Primary flow cytometric data (**d**) and frequencies of IFNγ-producing (**e**, **g**) or Granzyme B (GZMB)-producing (**f**) OT-I T cells in dLN and spleen, as determined after in vitro stimulation with IL12 + IL-18 (D-F) or OVA peptide (SIINFEKL) (**g**). **h** Experimental set up. CD45.1^+^ OT-I T cells were adoptively transferred into CD45.2^+^ recipient mice (*n* = 5 per group) that 1 day later received OVA-encoding Help or No Help pDNA vaccine. At day 50 after primary vaccination, mice were rechallenged with E7-encoding Help vaccine and injected with isotype control or neutralizing mAb to IL-12, 4 times, every 3 days. At day 10 after secondary vaccination, total dLN and spleen cells were incubated for 5 h with GolgiPlug (no cytokines or antigens were added). **i** Frequencies of IFNγ-producing CD45.1^+^ OT-I T cells isolated from indicated organs. **j** Representative flow cytometric analysis of cells isolated from spleen. Data are from one experiment representative of two experiments. Error bars indicate SD, *p < 0.05, **p < 0.01, ***p < 0.001 (unpaired two-tailed Student’s *t* test). Source data are provided as a Source Data file.

The transcriptome data from T_EM_ cells suggested that helped memory cells have increased sensitivity to IL-12 and IL-18, since the receptors for these cytokines were upregulated (Fig. 3f). It has been reported that combined stimulation with IL-12 and IL-18 can reactivate memory CD8^+^ T cells in an antigen non-specific manner, enabling them to exert effector functions^2,27,28^. We therefore tested whether help created this type of innate memory. For this purpose, we generated OVA-specific CD8^+^ memory T cells by adoptive transfer of OT-I T cells and vaccination with OVA-encoding Help or No Help vaccine (Fig. 4a). At day 100, the cell surface expression of both IL-18Rα chain (Fig. 4b) and IL-12Rβ chain (Fig. 4c) was significantly higher on helped than on helpless memory OT-I T cells in dLN, spleen and blood. To test the innate recall function, steady-state memory OT-I cells taken from dLN and spleen were stimulated in vitro with IL-12 and IL-18. The frequency of memory cells that could produce IFNγ (Fig. 4d, e) or Granzyme B (Fig. 4f) was significantly higher in the helped population as compared to the helpless population. This feature was characteristic for memory OT-I cells, as opposed to naïve cells. Also upon in vitro stimulation with cognate antigen (SIINFEKL), the frequency of IFNγ producing OT-I cells was higher in the helped memory population compared to the helpless or the naive populations (Fig. 4g).

We also tested in vivo whether help signals optimized memory CD8^+^ T cells for antigen-independent, innate responsiveness. For this purpose, OT-I T cells were transferred into recipient mice that were then vaccinated with OVA-encoding Help or No Help vaccine. After 50 days, these mice were vaccinated with HPV-E7-encoding Help vaccine (Fig. 4h). In this setting, the CD4^+^ T cell response is an antigen-specific memory response, since the same helper epitopes are present in the OVA- and E7-encoding DNA vaccines. The memory CD8^+^ T cells formed after OVA vaccination might be recalled in an antigen-independent manner, by cytokines such as IL-12 and IL-18 made by CD4^+^ T cell-activated DCs. To test the role of IL-12, neutralizing anti-IL-12 mAb was injected upon recall. At day 10 after secondary vaccination, the frequency of memory OT-I T cells that produced IFNγ upon non-cognate recall was significantly higher in the helped than in the helpless group (Fig. 4i, j). Moreover, treatment with neutralizing αIL-12 antibody significantly reduced IFNγ production by helped OT-I T cells, while not affecting the response of helpless cells (Fig. 4i, j). In conclusion, delivery of CD4^+^ T cell help during priming generated memory CD8^+^ T cells with innate-like recall capacities, being able to produce Granzyme B and IFNγ upon restimulation by cytokines, in the absence of cognate antigen.

### Helped memory CD8^+^ T cells are help-independent upon recall

**Fig. 5:**
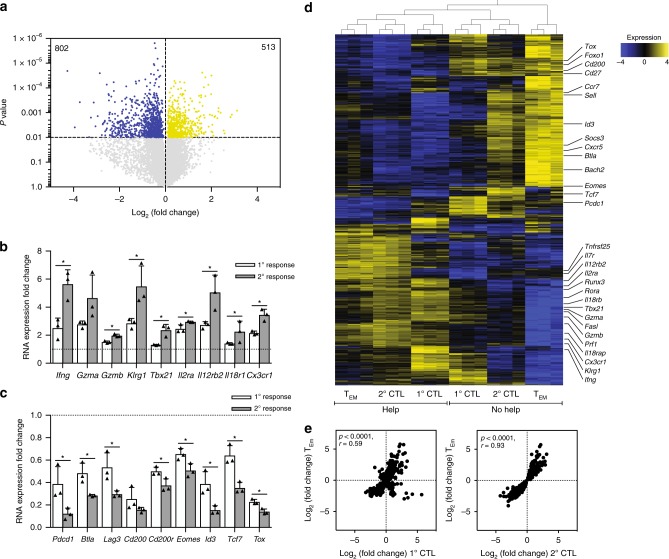
Helped memory CD8^+^ T cells express the Help signature genes after recall with MHC class I-restricted peptide only. Mice received primary vaccination with Help or No Help vaccine and were recalled at day 50 with No Help vaccine + LPS, as outlined in Fig. 1a. At day 10 of the secondary response, E7-specific CD8^+^ T cells were flow cytometrically isolated form the spleen (*n* = 3) and samples were processed for RNAseq. **a** Volcano plot depicting results of comparative transcriptome analysis of helped versus helpless secondary effectors. Blue and yellow dots indicate transcripts with significant differential expression between the two groups (*p* < 0.01) See also Supplementary Data 3. **b**, **c** Fold change in mRNA expression of a number of signature genes differentially expressed in the Help versus No Help setting of the primary response (1^o^), as determined before^20^ or the secondary response (2^o^), as determined here. **d** Hierarchical clustering of genes that were differentially expressed in the Help versus No Help groups in steady-state T_EM_ (Fig. 3), primary (1^o^) CTLs^20^ or secondary (2^o^) CTLs (**a**). Certain genes of known or suspected functional or diagnostic relevance are annotated. **e** Correlation between log_2_ fold changes in mRNA expression of genes differentially expressed in helped versus helpless T_EM_ cells and primary (1^o^) CTLs (left panel) or secondary (2^o^) CTLs (right panel). Error bars indicate SD, *p < 0.05 (unpaired two-tailed Student’s *t* test). Source data are provided as a Source Data file.

We have previously identified the molecular program instilled into primary CTL effectors by CD4^+^ T cell help^20^. Here, we examined which transcriptional program unfolded in memory CD8^+^ T cells after antigen rechallenge as a result of help delivered during priming. Mice that had been vaccinated with Help or No Help vaccine were rechallenged with No Help vaccine. At day 10 after rechallenge, E7-specific CD8^+^ T cells were isolated from the spleen and analyzed by transcriptomics. Statistical analysis of normalized transcript read counts indicated differential expression of 1315 genes (Fig. 5a, Supplementary Data 3). Of these, 391 genes were also differentially expressed as a result of help in the primary CTL response (Supplementary Data 4). We have previously described many of these transcripts as part of the signature of helped CTLs and showed for a number of these their importance in dictating the cytotoxic and migratory potential of CTLs in a tumor model^20^. Part of this signature were transcripts encoding cytotoxic effector molecules (*Ifng, Gzma, Gzmb*), the effector marker KLRG1 (*Klrg1*), transcription factor Tbet (*Tbx21*), receptors for IL-2, IL-12, and IL-18 (*Il2ra, Il12rb, Il18r*) and chemokine receptor CX3CR1 (*Cx3cr1*) that were all upregulated as a result of help (Fig. 5b). Moreover, transcripts encoding coinhibitory receptors PD-1, LAG3, BTLA, CD200(R) (*Pdcd1, Lag3, Btla, Cd200, Cd200r*) and transcription factors EOMES, ID3 TCF7, TOX (*Eomes, Id3, Tcf7, Tox*) that have all been connected to CTL dysfunction^31^ were similarly downregulated in “helped” CTLs in primary and secondary responses (Fig. 5c). Many differences in gene expression observed between Help and No Help conditions were more pronounced in the secondary response (Fig. 5b, c). Thus, CD8^+^ T cells that receive help during priming acquire the intrinsic ability to adopt a helped gene expression program after recall.

Next, we compared gene expression profiles of helped T_EM_ cells to those of helped primary and secondary effectors. Hierarchical clustering of differentially expressed genes revealed that expression profiles of helped secondary effectors were similar to those of T_EM_ cells that had received help during priming (Fig. 5d). Fold-changes of differentially expressed genes in the Help versus No Help setting were correlated between the T_EM_ population and primary effectors, and this correlation was even stronger between T_EM_ cells and secondary effectors (Fig. 5e). These findings suggest that helped secondary effectors are largely derived from helped T_EM_ cells.

Altogether, these data indicate that delivery of CD4^+^ T cell help during priming favor the formation of T_EM_ cells that maintain an imprint of help^20^ and can express the program characteristic of helped, more potent primary effectors upon recall with MHC class I restricted peptide only.

### Helped memory cells display epigenetic imprinting of help

**Fig. 6:**
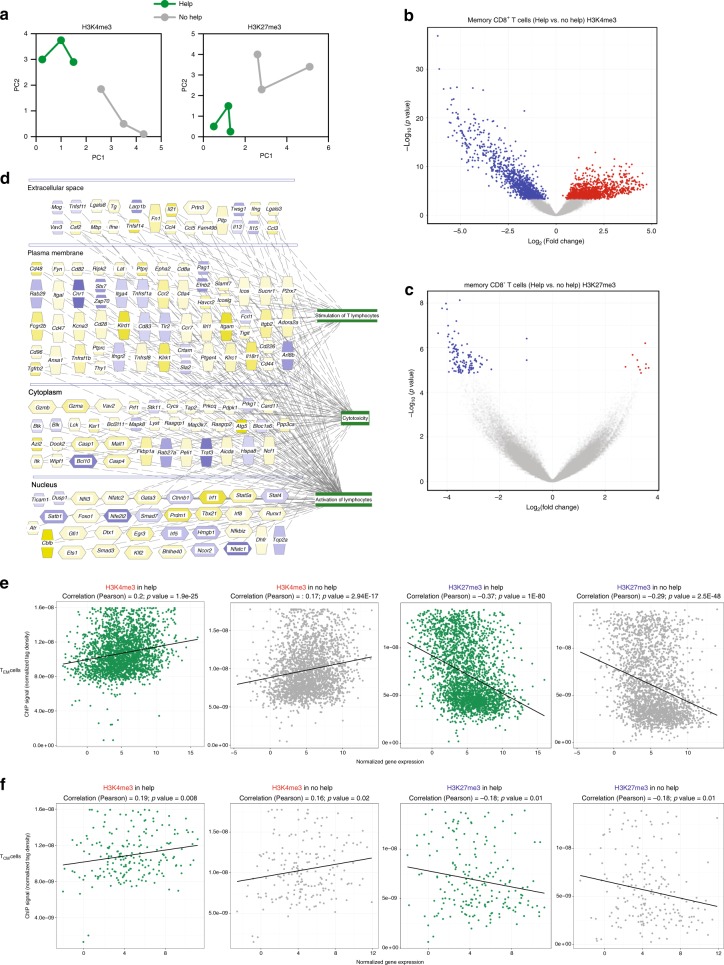
Help delivered during priming leaves an epigenetic imprint in steady-state memory cells. Mice (*n* = 3 per group) received Help or No Help vaccine on days 0, 3, and 6. At day 100, E7-specific memory CD8^+^ T cells were isolated from the spleens and subjected to ChIPseq analysis with antibodies against H3K4me3 and H3K27me3. **a** PCA plots depicting results of ChIPseq analysis. **b**, **c** Volcano plots depicting differentially modified regions (DMRs) as designated by H3K4me3 (**b**) or H3K27me3 (**c**) marks. Blue and red dots indicate DMRs with significant differential modifications (FDR < 0.05). **d** Molecules differentially modified by H3K4me3 in helped memory E7-specific CD8^+^ T cells, as assigned to functional categories and annotated for subcellular localization by IPA. **e**, **f** Correlation between differential mRNA expression in helped versus non-helped T_EM_ (**e**) and T_CM_ (**f**) cells and histone methylation in gene bodies and regions maximally 1 kb upstream from the TSS. Normalized tags of H3K4me3 and H3K27me3 are shown versus average normalized mRNA expression values in helped versus non-helped T_EM_ (**e**) and T_CM_ (**f**) cells (described in Fig. 5).

The data outlined above argue that memory CTLs “remember” that they have been helped during priming and are consequently help-independent after recall. We characterized the epigenetic states of helped and helpless steady-state memory CTLs to examine a potential epigenetic basis for this. Cells were isolated from spleen based on CD8 and H-2D^b^/E7_48-57_-tetramer staining at day 100 after vaccination. Given the low numbers of cells recovered, we used an ultra-low-input protocol for native chromatin immunoprecipitation (ULI-NChIP)^32^, with antibodies against H3K4me3 and H3K27me3. These two histone marks characterize permissive and repressed chromatin states that respectively favor and disfavor gene transcription^33^. After high throughput DNA sequencing, on average 54662 H3K4me3 and 104758 H3K27me3 peaks (FDR < 0.01) were identified in helped and helpless steady-state memory CTL samples. Principal component analysis (PCA) showed that these CTL populations had distinct profiles for both epigenetic marks (Fig. 6a).

We identified consensus peaks in chromatin of helped or helpless memory CTLs that contained significantly different amounts of H3K4me3 and H3K27me3 deposition (FDR < 0.05) (Fig. 6b, c, Supplementary Data 5, 6). These differentially modified regions (DMRs) were annotated to the nearest transcriptional start sites (TSSs). Genes that were significantly enriched in H3K4me3 marks in helped memory CTLs were assigned by IPA to the top functional categories (z-score > 2) “stimulation of T lymphocytes”, “cytotoxicity” and “activation of lymphocytes” (Fig. 6d). The epigenetic marks were congruent with the previously defined help gene expression program^20^ being opened in helped memory cells, as indicated by an open configuration of *Ifng, Gzma Gzmb, Prf1* genes encoding cytotoxic effector molecules and transcription factors *Tbx21 and Prdm1* (Fig. 6d). H3K4me3 deposition positively correlated with differential mRNA expression in helped versus helpless steady-state T_EM_ and T_CM_ cells, while H3K27me3 deposition negatively correlated with differential mRNA expression in these populations (Fig. 6e, f). These data argue that CD4^+^ T cell help brings about epigenetic changes that allow memory CTLs to display a helped phenotype at steady state and after recall in the absence of help signals.

## Discussion

Therapeutic vaccines against cancer or pathogens aim to raise CTL responses. A historical disconnect between the fields of vaccinology and immunology has reportedly delayed the development of effective synthetic T cell inducing vaccines^34,35^. Vaccinology currently focuses on incorporating innate DC-activating signals into such vaccines^35^. However, helper epitopes must also be included^36^. Moreover, it should be ensured that both CTL- and helper epitopes reach the appropriate DCs^37,38^. Recent intravital imaging studies showed CD4^+^ T cell help is transmitted from a CD4^+^ T cell to the CD8^+^ T cell by a lymph node-resident cDC1^18,39^. This event occurs during a second step of priming, after the naïve CD8^+^ and CD4^+^ T cells have been activated by a (migratory) conventional (c)DC1, or cDC2, respectively, that has been stimulated by innate signals^17,18,39,40^.

The help signals lead to upregulation of IL-12 or IL-15 and the costimulatory ligands CD80/86 and CD70 on the LN-resident cDC1^15,17^. As a result, the CD8^+^ T cell receives costimulatory input via CD28, CD27 and cytokine receptors that lead to optimal CTL effector differentiation^17,20^. The consensus has been that primary CTL responses benefit from help only when innate DC-activating signals are lacking^41^. However, we have recently demonstrated by transcriptome analysis that CD4^+^ T cell help optimizes the quality of the primary CTL response after both DNA vaccination and virus infection, by similar molecular mechanisms^20^.

CD4^+^ T cell help optimizes many intrinsic properties of the CTL, including its cytotoxic, migratory and invasive capacities^20^. We determined that the second step of priming is a “checkpoint” for CTL functionality, since CTLs raised in the absence of help had an overall dysfunctional phenotype, characterized by low expression of cytotoxic effector molecules and increased expression of PD-1, BTLA and other coinhibitory molecules at the cell surface, which prevented them from killing target cells. The impact of help on the primary CTL effector capabilities as demonstrated in our study was not vaccination model-specific, but had a general validity. We showed in the same study that the helped versus helpless gene expression profile could also be identified by transcriptomics in CTLs raised against the acute virus strain LCMV-Armstrong. Moreover, the same traits were identified by gene array in a different vaccination model by other researchers^42^.

Pioneering studies showed that CD4^+^ T cell help delivered during priming promotes formation and maintenance of the memory CD8^+^ T cell pool, but this was not specified to memory T cell subsets at the time^43,44^. We show here that CD4^+^ T cell help primarily increases the steady-state CD8^+^ T_EM_ cell pool and has a relatively minor effect on the T_CM_ pool. IL-15 receptor signaling upregulates anti-apoptotic Bcl-2 proteins and has been implicated in the survival of memory CD8^+^ T cells^45,46^. We found that CD4^+^ T cell help increased expression of the IL-2Rβ (CD122) chain on steady-state CD8^+^ T_CM_ cells and not on T_EM_ cells. Accordingly, IL-15 drove the maintenance of helped T_CM_ cells, but not T_EM_ cells, in agreement with earlier findings^46^. This result suggests, in agreement with another report^47^, that T_EM_ cells rely on other, yet undefined, help-dependent mechanisms for their long-term survival.

Helped T_EM_ cells had upregulated transcripts encoding many molecules implicated in cytotoxic functions, including Granzyme A and B, Perforin, Fas ligand, Trail and IFNγ. Increased “surveillance” activity was also suggested by upregulation of transcripts encoding inflammasome components Pycard (Asc), Caspase-1 and NOD-like receptors that regulate IL-1 production^29^. Memory CD8^+^ T cells are known to acquire functional properties characteristic of innate immune cells, allowing them to protect against pathogens unrelated to the ones responsible for priming^27,28^. In specific cellular niches, these pathogens would evoke the production of cytokines that locally activate memory CD8^+^ T cells, reportedly to produce IFNγ^2^. In memory CD8^+^ T cells, a pool of presynthesized *Ifng* mRNA is available that is freed for rapid translation into protein upon recall^12^. A combination of IL-12 and IL-18 was found to be particularly potent in inducing antigen-independent IFNγ production by memory CD8^+^ T cells^27^. We accordingly found that helped memory CD8^+^ T cells responded to combined stimulation by IL-12 and IL-18 by IFNγ and Granzyme B protein production. We did not specify in these experiments whether T_EM_ cells were responsible for this response, but this is likely, because T_EM_ (and not T_CM_) cells showed help-dependent upregulation of IL-12Rβ and IL18Rα chains, and previous studies have demonstrated that T_EM_ cells are more responsive to IL-12 than T_CM_ cells^48^. We also show that helped memory cells responded in vivo—in an IL-12-dependent manner—to recall by non-cognate antigen. The intra-epidermal vaccination procedure presumably evokes a cytokine alert in the keratinocytes where the vaccine DNA is expressed^37^, which explains this response. The ability of helped T_EM_ cells to mount more efficient innate recall responses than helpless T_EM_ cells may not only be due to increased expression of specific cytokine receptors, but also to other cell-intrinsic alterations, which we show here to be many.

We performed epigenetic analysis on total steady-state CD8^+^ memory T cells, since the cell input was too small to do this for separate T_EM_ and T_CM_ subsets. However, the genes that were epigenetically altered as a result of help signals were differentially expressed in T_EM_ cells and not in T_CM_ cells. This result argues that primarily the T_EM_ cell pool was epigenetically modified as a result of help signals delivered during priming. The genes that were epigenetically modified to facilitate transcription fell in the functional categories “stimulation/activation of lymphocytes” and “cytotoxicity”, again indicating a higher alert state of helped T_EM_ cells. The epigenetic marks correlated with gene expression. Helped T_EM_ cells were transcriptionally distinct from either helped or helpless T_CM_ cells (Fig. 3c), indicating that they constituted cells with a unique differentiation state.

Once helped during priming, memory CD8^+^ T cells no longer require help to undergo secondary expansion upon recall with cognate antigen^43,44,49,50^. In our vaccination model, TLR stimulation by LPS was required to recall helped memory CD8^+^ T cells with the No Help vaccine. Likely, this promoted antigen presentation and/or other functions of DCs that play a key role in initiating secondary T cell responses^51^. Helped memory CD8^+^ T cells were superior over their helpless counterparts in secondary expansion and cytotoxicity, due to cell-intrinsic mechanisms. This memory programming of CD8^+^ T cells by CD4^+^ T cell help has been described many years ago^13^, but we identify here its molecular basis. Helped memory CTLs expressed, after recall in absence of help signals, the signature gene set identified in helped primary CTLs. The gene signature characteristic for helped secondary effectors was in large part already present in steady-state helped T_EM_ cells (Fig. 5d). These data, together with the epigenetic data we provide, argue that CD8^+^ T cells acquire, as a result of help signals delivered during priming, a unique T_EM_ cell differentiation program that allows them to perform specific surveillance functions at steady-state and to display a helped gene expression program after recall in the absence of help signals.

## Methods

### Mice

Gender-matched, 7–9-week-old mice were maintained under specific-pathogen free conditions, in accordance with national regulations for animal testing and research. Ethical approval for this study was granted by the institutional Experimental Animal Committee (DEC) of the Netherlands Cancer Institute. C57BL/6JRj mice were purchased from Janvier Laboratories. CD45.1^+^ and OT-I;CD45.1^+^ mice were bred in-house.

### Vaccination

For vaccination, we used the HELP-E7SH and E7SH DNA vaccines^21,22^. OVA DNA vaccines were generated by the C-terminal addition of the OVA_257-264_ coding sequence to the HELP-E7SH and E7SH DNA constructs. For DNA “tattoo” vaccination, the hair on a hind leg was removed using depilating cream (Veet) on day −1. On days 0, 3, and 6, mice were anesthetized, and 15 μl of a 2 mg/ml DNA solution in 10 mM Tris-HCl, 1 mM EDTA, pH 8.0, was applied to the hairless skin with a Permanent Make Up tattoo machine (Amiea, MT Derm GmbH), using a sterile disposable nine-needle bar with a needle depth of 1 mm and oscillating at a frequency of 100 Hz for 45 s. For rechallenge (at day 50 after primary vaccination), the hair removal step was repeated, and mice received a single DNA tattoo and an i.p. injection with 5 μg LPS from E. coli 055:B5 (Sigma) in 100 μl Hank’s Buffered Salt Solution (HBSS) on the next day (day 0, rechallenge).

### Antibody treatments

Neutralizing mAb to IL-15 (GRW15PLZ, Thermo Fisher Scientific) was injected i.p as described^52^ at 5 μg per injection, three times per week from day 20 to day 50 after primary vaccination. Neutralizing mAb to IL-12p75 (R2-9A5, Bio X Cell) or isotype control mAb (LTF-2, Bio X Cell) were injected i.p. at 500 μg per mouse in 100 μl HBSS on days 0, 3, 6, and 9.

### Tissue preparation and flow cytometry

Peripheral blood cells were obtained by tail bleeding; spleen, inguinal lymph nodes, and bone marrow were passed through a 70 μm cell strainer (BD Falcon). After erythrocyte lysis, cell suspensions were washed and stained with relevant mAbs and phycoerythrin-conjugated H-2D^b^/E7_49-57_ or H-2K^b^/OVA_257-264_ tetramers. For intracellular staining of transcription factors, cells were fixed and permeabilized with the FOXP3/transcription factor staining buffer set (Thermo Fisher Scientific). Flow cytometry was performed using the LSRFortessa (BD Biosciences) and data were analyzed with FlowJo software (Tree Star Inc.). Live cells were selected based on near-infra red dye (Life Technologies) exclusion. Antibody details are listed in Supplementary Data 8.

### Isolation of antigen-specific CD8^+^ T cells

At day 50 or 100 after primary vaccination, or at day 10 after secondary vaccination, mice were sacrificed, spleens were isolated and passed through 70 μm cell strainer (BD Falcon). After erythrocyte lysis, cell suspensions were washed, stained with anti-CD8α mAb (53-6.7, eBioscience), anti-CD44 (IM7, eBioscience) and anti-CD62L (MEL-14, BD Pharmingen) and PE-conjugated H-2D^b^/E7_49-57_ or H-2K^b^/OVA_257-264_ tetramers and sorted on a BD FACSAria Fusion cell sorter. Live cells were selected based on propidium iodide exclusion. All samples were maintained at 4^o^C for the duration of the sort, and purity was at 95%-98% for all samples. Isolated cells were subsequently used for in vivo, in vitro, RNAseq or ChIPseq assays.

### In vivo cytotoxicity assay

Splenocytes from naive mice were labeled ex vivo with 0.1 μM carboxyfluorescein succinimidyl ester (CFSE; Life Technologies) and pulsed with 5 μM E7_49-57_ peptide (specific target) or labeled with 1 μM CFSE and left unpulsed (control). Subsequently, 5 × 10^6^ cells of each population were injected retro-orbitally (r.o.) in the same recipient mouse, and 16 h later, spleens were analyzed by flow cytometry. Percent killing was calculated as follows: 100 − ([(% specific targets in vaccinated recipients/% control targets in vaccinated recipients)/(% specific targets in control recipients/% control targets in control recipients)] × 100).

### In vitro cytotoxicity assay

To create target cells, splenocytes from naïve mice were labeled with 0.1 μM Cell Trace Violet (CTV; Thermo Fisher Scientific) and pulsed with 5 μM E7_49-57_ peptide (specific target) or labeled with 1 μM CFSE (Thermo Fisher Scientific) and left unpulsed (control target). Subsequently, 5000 ex vivo isolated H-2D^b^/E7_49-57_-specific CD8^+^ T cells were mixed with equal numbers of the two target cell populations in 1:1 ratio and incubated at 37 °C, 5% CO_2_ for 12 h. Cells were then analyzed by flow cytometry. Percent killing was calculated as follows: 100 − ([(% specific targets /% control targets)/(% control targets in absence of effectors/% specific targets in absence of effectors)] × 100).

### Adoptive transfer experiments

E7-specific memory CD8^+^ T cells that had been flow cytometrically sorted ex vivo with MHC tetramers were injected at 1 × 10^5^ cells per mouse r.o. into naïve WT C57BL/6 mice. Naïve OT-I CD8^+^ T cells that carry a transgenic Vα2/Vβ5 TCR specific for H-2K^b^/OVA_257-264_ were purified to 95% homogeneity form spleens of OT-1;CD45.1^+^ mice with the CD8^+^ T cell Isolation Kit (Miltenyi Biotec) and injected r.o. into WT C57BL/6 mice at 1.5 × 10^5^ cells per mouse. One day later, mice received DNA vaccination, unless indicated otherwise.

### In vitro restimulation assay

Prior to cytokine detection with the BD Cytofix/Cytoperm Kit (BD Biosciences), cells were incubated for 16 h with or without E7_49-57_ peptide (RAHYNIVTF) or for 5 h with 5 ng/ml recombinant IL-12 p70 (Thermo Fisher Scientific) and 10 ng/ml IL-18 (MBL) in the presence of GolgiPlug in IMDM with 8% FCS or GolgiPlug in medium alone.

### RNA sequencing

For isolation of T_CM_ and T_EM_ cells, H-2D^b^/E7_49-57_-specific CD8^+^ T cells were flow cytometrically sorted based on anti-CD8, anti-CD62L, anti-CD44 and tetramer staining from three mice per experimental group. Cells were resuspended in Trizol (Ambion Life Technologies) and total RNA was extracted according to the manufacturer’s protocol. Quality and quantity of the total RNA was assessed by the 2100 Bioanalyzer using a Nano chip (Agilent). Only RNA samples having an RNA Integrity Number (RIN) > 8 were subjected to library generation. Strand-specific cDNA libraries were generated using the TruSeq Stranded mRNA sample preparation kit (Illumina) according to the manufacturer’s protocol. The libraries were analyzed for size and quantity of cDNAs on a 2100 Bioanalyzer using a 7500 chip (Agilent), diluted and pooled at 10 nM in multiplex sequencing pools. The libraries were sequenced as 50 base single reads in two lanes on a HiSeq2500 with V3 chemistry (Illumina).

### Chromatin immunoprecipitation sequencing

Between 6 and 9 × 10^4^ E7-specific memory CD8^+^ T cells per sample were subjected to ULI-NChIP procedure^32^. For each immunoprecipitation, 0.25 μg of αH3K4me3 mAb (#9272, Cell Singaling Technology), or 0.25 μg of αH3K27me3 mAb (C15410069, Diagenode) was used. Input DNA for each sample was used as a control. Quality control metrics of Chromatin immunoprecipitation sequencing (ChIPseq) experiment are summarized in Supplementary Data 7.

### RNAseq analysis

Illumina adapter sequences were trimmed from Fastq files using trimmomatic (v. 0.36) with 4-base wide sliding window, to trim reads when the average quality per base drops below 15. Any reads remaining below ≤36 bases long were filtered out. Adapter-trimmed files were aligned to GRCm38.77 with STAR (v. 2.5.3a). Using the genes (GRCm38.77.gtf), HTSeq-count was used in intersection-strict mode for exon feature type to generate a count matrix for all samples. Differential expression analysis was performed using Qlucore Omics Explorer (v. 3.4). Genes with less than 5 reads in at least one of the samples were discarded. Mapping quality threshold was set to 10. TNM normalization method was applied. Differential expression with two-group comparison was considered significant at *P* < 0.01. GSEA was performed using Qlucore Omics Explorer. Functional classification of the genes and identification of enriched signaling pathways was performed using IPA software (Qiagen).

### ChIPseq analysis

Single-end fastq files were aligned to mm10 (GrCm38.77) using bwa (v. 0.7.17). Resulting alignments were filtered for mapping quality > MQ20 using samtools (v. 1.5). Peak calling for H3K27me3 ChIP-seq was performed using MACS2 (v. 2.1.1) with detection for broad peaks using input for normalization. For H3K4me3 we performed narrow peak calling using MACS (v. 2.1.1) with input normalization. For differential binding, we used the R package Diffbind (v. 2.2.1) to determine differentially bound peaks found in all replicates for “No Help” vs “Help” with the EdgeR method. We annotated the bound regions using BEDTools (v. 2.17.0) closest Bed reporting the first feature against a file of genes and locations determined from GRCm38.77 gtf file selecting for “gene_name”. To show correlation of ChIP-sequencing tags versus mRNA expression, we merged the replicates for each cell type using samtools and counted reads in the gene body including 1000 bp upstream from the TSS from the merged alignments. Data were normalized by dividing by region size and library read count. For each gene, normalized tags are shown versus average normalized gene expression values for “No Help” vs “Help” cell types of both ChIP-sequencing marks (H3K27me3 and H3K4me3). Table for quality control metrics was produced with the R package ChIPQC for ChIP-seq experiments. To generate PCA plots, reads were counted in 10 kb bins across the genome for each sample using deepTools (v. 2.5.7) multiBamSummary bins function. The binned counts were output to a table and log transformed. PCA was carried out on these data using R function prcomp.

### Statistical analysis

Data was analyzed with GraphPad Prism software using unpaired two-tailed Student’s t or one-way ANOVA test. Error bars in figures indicate SD. A *P* value < 0.05 was considered statistically significant; *P < 0.05, **P < 0.005, ***P < 0.001 and ****P < 0.0001.

### Reporting summary

Further information on research design is available in the Nature Research Reporting Summary linked to this article.

## Supplementary information

Supplementary Information

Reporting Summary

Description of Additional Supplementary Files

Supplementary Data 1

Supplementary Data 2

Supplementary Data 3

Supplementary Data 4

Supplementary Data 5

Supplementary Data 6

Supplementary Data 7

Supplementary Data 8

## Data Availability

RNAseq and ChIPseq data have been deposited in the GEO database under the accession code GSE118160 [https://www.ncbi.nlm.nih.gov/geo/query/acc.cgi?acc = GSE118160]. All other data supporting findings in this study are available from the corresponding author upon request.

## Source data

Source Data
