# Supplementary material for: CD4+ T cell help creates memory CD8+ T cells with innate and help-independent recall capacities
Source: Nat Commun. 2019 Dec 4;10:5531. doi: 10.1038/s41467-019-13438-1 (PMC6892909; doi:10.1038/s41467-019-13438-1)
Supplement: Supplementary file 1 — Supplementary Information [file 41467_2019_13438_MOESM1_ESM.pdf]

Supplementary Information

$CD4^+$ T cell help creates memory $CD8^+$ T cells with innate and help-independent recall capacities

Ahrends *et al.*

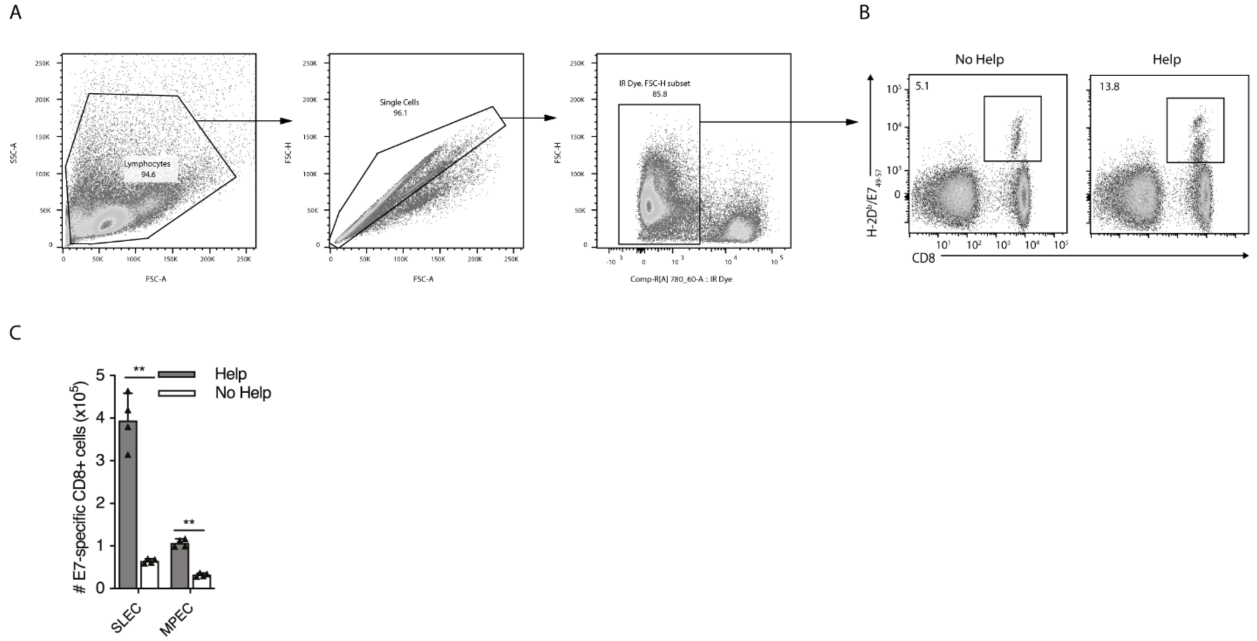

Supplementary Figure 1. (A, B, C) Mice (n=4 per group) were vaccinated as described for Figure 1. (A) Representative flow cytometry gating strategy. 3 initial steps of the gating strategy were used in all experiments. (B) Representative flow cytometric plots indicating in the boxes H-2D^b/E7$_{49-57}$ (E7)-specific CD8$^+$ T cells as measured in the spleen at the peak of the primary response (day 10). (B) Absolute numbers (#) of E7-specific CD8$^+$ T cells among CD127$^-$KLRG1$^+$ SLECs and CD127$^+$KLRG1$^-$ MPECs, as measured in the spleen at day 10 after primary vaccination. Error bars indicate SD, **p < 0.01 (unpaired two-tailed Student’s t test). Source data are provided as a Source Data file.

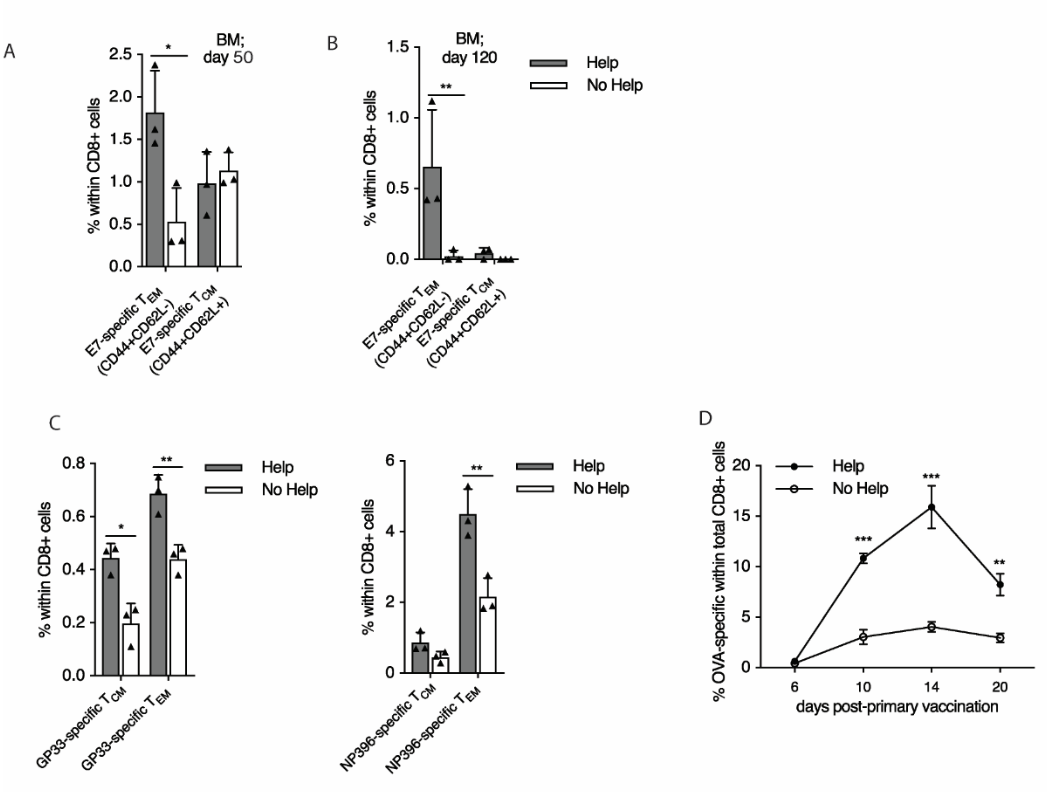

Supplementary Figure 2. (A,B) Mice (n=5 per group) received Help or No Help vaccine on days 0, 3 and 6 and were analyzed on days 50 or 120. Quantification of frequencies of E7-specific $CD44^+CD62^-$ T_{EM} and $CD44^+CD62^+$ T_{CM} phenotype $CD8^+$ T cells determined in BM at day 50 (A) and day 120 (B). (C) Mice were infected with 10^5 PFU of LCMV Armstrong i.p. At day 2 before and days 2 and 4 after the infection, mice were injected i.v. with depleting anti-CD4 mAb (GK1.5) at 200 μg per mouse (No Help) or with isotype control antibody (Help). Graphs indicate frequencies of GP33- and NP396-specific T_{EM} ($CD62L^-CD44^+$) and T_{CM} ($CD62L^+CD44^+$) cells at day 50 post-infection. (D) $CD45.1^+$ OT-I T cells were adoptively transferred into $CD45.2^+$ recipient mice (n=5 per group) that 1 day later received OVA-encoding Help or No Help pDNA vaccine. Depicted is the percentage of H-$2K^b$/$OVA_{257-264}$ tetramer$^+$ cells among total $CD8^+$ T cells in the blood at the indicated days after the vaccination. Error bars indicate SD, *p < 0.05, **p < 0.01, ***p < 0.001 (unpaired two-tailed Student's t test). Source data are provided as a Source Data file.
