# Supplementary material for: CD4+ T cell help creates memory CD8+ T cells with innate and help-independent recall capacities
Source: Nat Commun. 2019 Dec 4;10:5531. doi: 10.1038/s41467-019-13438-1 (PMC6892909; doi:10.1038/s41467-019-13438-1)
Supplement: Supplementary file 2 — Reporting Summary [file 41467_2019_13438_MOESM2_ESM.pdf]

nature research

Corresponding author(s): Jannie Borst

Reporting Summary

Nature Research wishes to improve the reproducibility of the work that we publish. This form provides structure for consistency and transparency in reporting. For further information on Nature Research policies, see Authors & Referees and the Editorial Policy Checklist.

Statistical parameters

When statistical analyses are reported, confirm that the following items are present in the relevant location (e.g. figure legend, table legend, main text, or Methods section).

n/a	Confirmed	
☐	☒	The exact sample size (*n*) for each experimental group/condition, given as a discrete number and unit of measurement
☐	☒	An indication of whether measurements were taken from distinct samples or whether the same sample was measured repeatedly
☐	☒	The statistical test(s) used AND whether they are one- or two-sided<br>*Only common tests should be described solely by name; describe more complex techniques in the Methods section.*
☐	☒	A description of all covariates tested
☒	☐	A description of any assumptions or corrections, such as tests of normality and adjustment for multiple comparisons
☐	☒	A full description of the statistics including central tendency (e.g. means) or other basic estimates (e.g. regression coefficient) AND variation (e.g. standard deviation) or associated estimates of uncertainty (e.g. confidence intervals)
☐	☒	For null hypothesis testing, the test statistic (e.g. *F*, *t*, *r*) with confidence intervals, effect sizes, degrees of freedom and *P* value noted<br>*Give P values as exact values whenever suitable.*
☒	☐	For Bayesian analysis, information on the choice of priors and Markov chain Monte Carlo settings
☐	☒	For hierarchical and complex designs, identification of the appropriate level for tests and full reporting of outcomes
☒	☐	Estimates of effect sizes (e.g. Cohen's *d*, Pearson's *r*), indicating how they were calculated
☐	☒	Clearly defined error bars<br>*State explicitly what error bars represent (e.g. SD, SE, CI)*

Our web collection on statistics for biologists may be useful.

Software and code

Policy information about availability of computer code

Data collection	Flow cytometry data were acquired with BD FACSDIVA software (v8).<br>RNAseq and ChIPseq data were collected on Illumina HighSeq2500 sequencer.
Data analysis	RNAseq data were analyzed with trimmomatic (v 0.36), STAR (v. 2.5.3a) and Qlucore Omics Explorer (v 3.4), BiNGO plugin (v 3.0.3) in Cytoscape (v 3.4.0) and Ingenuity Pathway Analysis software.<br>ChIPseq data were analyzed with the use of following packagaes: bwa (v 0.7.17), samtools (v 1.5), MACS2 (v 2.1.1), Diffbind (v 2.2.1), BEDTools (v 2.17.0), deepTools (v 2.5.7) and EaSeq.<br>Statistical analysis was performed with GraphPad Prism software (v 7).<br>Flow cytometry data were analyzed with FlowJo software (v 10).

For manuscripts utilizing custom algorithms or software that are central to the research but not yet described in published literature, software must be made available to editors/reviewers upon request. We strongly encourage code deposition in a community repository (e.g. GitHub). See the Nature Research guidelines for submitting code & software for further information.

Data

Policy information about availability of data

All manuscripts must include a data availability statement. This statement should provide the following information, where applicable:

- Accession codes, unique identifiers, or web links for publicly available datasets
- A list of figures that have associated raw data
- A description of any restrictions on data availability

RNAseq data and ChIPseq data are available in the GEO database under accession code GSE118160.
All other data supporting the findings in this study are available from the corresponding author upon request.

Field-specific reporting

Please select the best fit for your research. If you are not sure, read the appropriate sections before making your selection.

☒ Life sciences ☐ Behavioural & social sciences ☐ Ecological, evolutionary & environmental sciences

For a reference copy of the document with all sections, see nature.com/authors/policies/ReportingSummary-flat.pdf

Life sciences study design

All studies must disclose on these points even when the disclosure is negative.

Sample size	G*Power software was used to predetermine the sample sizes in all animal experiments with guidelines based on previous experience with the experimental protocols.
Data exclusions	No data points were excluded from data sets.
Replication	All the experimental data provided are based on biological replicates and are representative of at least 2 independent experiments (with the exception of RNAseq and ChIPseq experiments, performed only once)
Randomization	For animal work, mice were randomly assigned to experimental groups.
Blinding	For some vaccination experiments, the investigators were blinded to the type of the vaccine used. For most other experiments, the investigators were not blinded to group allocation.

Reporting for specific materials, systems and methods

Materials & experimental systems

n/a	Involved in the study	
☒	☐	Unique biological materials
☐	☒	Antibodies
☒	☐	Eukaryotic cell lines
☒	☐	Palaeontology
☐	☒	Animals and other organisms
☒	☐	Human research participants

Methods

n/a	Involved in the study	
☐	☒	ChIP-seq
☐	☒	Flow cytometry
☒	☐	MRI-based neuroimaging

Antibodies

Antibodies used	Following antibodies were used: eBioscience: CD103 (clone:E27), CD127 (clone: A7R34), CD44 (clone: IM7), CD45.1 (clone: A20), CD8 (clone: 53-6.7), IL-18Ra (clone: P4TUNYA), PRF1 (clone:eBioOMAK-D), T-BET (clone: eBio4B10). BioLegend: CD45.2 (clone: 104), IL-15Rb (clone: TM-b1), KLRG1 (clone: 2F1). BD Pharmigen: CD62L (clone: MEL-14), CD8 (clone: 53-6.7), IFNγ (clone: XMG1.2), TNF (clone: TN3-19.12). Enzo Life Sciences: GZMB (clone: CLB-GB11). RnD Systems: IL-12Rb (FAB1998F).
Validation	All antibodies were validated by the manufacturers and by our own and colleagues' labs where they are used extensively.

Animals and other organisms

Policy information about studies involving animals; ARRIVE guidelines recommended for reporting animal research

Laboratory animals

Only mice were used in this study and groups were age- and sex-matched for all experiments. In general female mice at 7-9 weeks were used throughout. C57BL/6JRj mice were purchased from Janvier Laboratories. CD45.1+ and OT-I;CD45.1+ mice were bred in-house. Mice were maintained under specific-pathogen free conditions at the Netherlands Cancer Institute, in accordance with national guidelines as approved by the institutional Experimental Animal Committee (DEC)

Wild animals

n/a

Field-collected samples

n/a

ChIP-seq

Data deposition

☒ Confirm that both raw and final processed data have been deposited in a public database such as GEO.

☒ Confirm that you have deposited or provided access to graph files (e.g. BED files) for the called peaks.

Data access links
May remain private before publication.

https://www.ncbi.nlm.nih.gov/geo/query/acc.cgi?acc=GSE118160

Files in database submission

Raw data:
Help 1 H3K4me3.fastq.gz
Help 1 H3K27me3.fastq.gz
Help 1 input.fastq.gz
Help 2 H3K4me3.fastq.gz
Help 2 H3K27me3.fastq.gz
Help 2 input.fastq.gz
Help 3 H3K4me3.fastq.gz
Help 3 H3K27me3.fastq.gz
Help 3 input.fastq.gz
No Help 1 H3K4me3.fastq.gz
No Help 1 H3K27me3.fastq.gz
No Help 1 input.fastq.gz
No Help 2 H3K4me3.fastq.gz
No Help 2 H3K27me3.fastq.gz
No Help 2 input.fastq.gz
No Help 3 H3K4me3.fastq.gz
No Help 3 H3K27me3.fastq.gz
No Help 3 input.fastq.gz
Help_62neg_001.bam
Help_62neg_002.bam
Help_62neg_003.bam
Help_62pos_001.bam
Help_62pos_002.bam
Help_62pos_003.bam
NoHelp_62neg_001.bam
NoHelp_62neg_002.bam
NoHelp_62neg_003.bam
NoHelp_62pos_001.bam
NoHelp_62pos_002.bam
NoHelp_62pos_003.bam
Processed data (BED files):
4500_7_NH1_K4_CGCTGATC_S14_L005_R1_001.sort.mq20.sort_v2_peaks.narrowPeak
4500_8_NH2_K4_ACAAGCTA_S15_L005_R1_001.sort.mq20.sort_v2_peaks.narrowPeak
4500_9_NH3_K4_CTGTAGCC_S7_L004_R1_001.sort.mq20.sort_v2_peaks.narrowPeak
4500_10_H1_K4_AGTACAAG_S8_L004_R1_001.sort.mq20.sort_v2_peaks.narrowPeak
4500_11_H2_K4_AACAACCA_S16_L005_R1_001.sort.mq20.sort_v2_peaks.narrowPeak
4500_12_H3_K4_AACCGAGA_S17_L005_R1_001.sort.mq20.sort_v2_peaks.narrowPeak
4500_13_NH1_K27_AACGCTTA_S9_L004_R1_001.sort.mq20.sort_v2_peaks.broadPeak
4500_14_NH2_K27_AAGACGGA_S10_L004_R1_001.sort.mq20.sort_v2_peaks.broadPeak
4500_15_NH3_K27_AAGGTACA_S18_L005_R1_001.sort.mq20.sort_v2_peaks.broadPeak
4500_16_H1_K27_ACACAGAA_S19_L005_R1_001.sort.mq20.sort_v2_peaks.broadPeak
4500_17_H2_K27_ACAGCAGA_S11_L004_R1_001.sort.mq20.sort_v2_peaks.broadPeak
4500_18_H3_K27_ACCTCCAA_S20_L005_R1_001.sort.mq20.sort_v2_peaks.broadPeak

Genome browser session
(e.g. UCSC)

https://genome.ucsc.edu/cgi-bin/hgTracks?
hgS_doOtherUser=submit&hgS_otherUserName=Tesa&hgS_otherUserSessionName=ahrends_et_al_2018

Methodology

Replicates	3 biological replicates were used for each experimental group. Each sample was sequenced after the immunoprecipitation with a specific antibody or as an 'input control'.
Sequencing depth	Read length: 65bp, single-end.<br>Number of reads for each sample:<br>NH1_H3K4me3 1381377<br>NH2_H3K4me3 1365344<br>NH3_H3K4me3 1245361<br>H1_H3K4me3 1473058<br>H2_H3K4me3 1442147<br>H3_H3K4me3 1633685<br>NH1_H3K27me3 1526509<br>NH2_H3K27me3 1517810<br>NH3_H3K27me3 1653020<br>H1_H3K27me3 1688909<br>H2_H3K27me3 1514747<br>H3_H3K27me3 1631541
Antibodies	aH3K27me3 mAb (C15410069, Diagenode) and aH3K4me3 mAb (#9272, Cell Singaling Technology).
Peak calling parameters	Single-end fastq files were aligned to mm10 (GrCm38.77) using bwa (v. 0.7.17). Resulting alignments were filtered for mapping quality > MQ20 using samtools version 1.5. Peak calling for H3K27me3 ChIP-seq was performed using MACS2 (v. 2.1.1) with detection for broad peaks using input for normalization. For H3K4me3 we performed narrow peak calling using MACS (v. 2.1.1) with input normalization.
Data quality	Table for quality control metrics was produced with the R package ChIPQC for ChIP-seq experiments and can be found in Table S5, including Peaks = number of peaks called in the sample, Reads = reads in the library, Dup% = number of duplicate reads, ReadL = read length, FragL = fragment length, RelCC = relative cross-coverage score, SSD = squared sum of deviations, RiP% = percent reads in peaks.
Software	bwa (v. 0.7.17)<br>samtools (v. 1.5)<br>MACS2 (v. 2.1.1)<br>Diffbind (v. 2.2.1)<br>BEDTools (v. 2.17.0)<br>deepTools (v. 2.5.7)

Flow Cytometry

Plots

Confirm that:

☒ The axis labels state the marker and fluorochrome used (e.g. CD4-FITC).

☒ The axis scales are clearly visible. Include numbers along axes only for bottom left plot of group (a 'group' is an analysis of identical markers).

☒ All plots are contour plots with outliers or pseudocolor plots.

☒ A numerical value for number of cells or percentage (with statistics) is provided.

Methodology

Sample preparation	Blood, lymph nodes, spleens and skin were collected and processed using standard methods as detailed in Materials and Methods section.
Instrument	LSRFortessa II (BD Biosciences)
Software	BD FACSDIVA software (v 8) and Flowjo software (v 10).
Cell population abundance	For cell sorting experiments between 10,000 - 100,000 cells were collected for each replicate.<br>For FACS analysis: the population abundance varied in different tissues and in different analysis - the relevant cell abundance is indicated within the results section.
Gating strategy	Flow cytometric analysis focused on identifying the frequencies of memory or activated CD8 T cells expressing a specific marker. In all experiments, cells were first gated on singlets (FSC-H vs FSC-A) before setting a lymphocyte gate based on SSC-A vs FSC-A. Dead cells were excluded from the analysis by using a Live/Dead IR dye (Invitrogen). Antigen-specific CD8 T cells were gated as CD8+ and tetramer+ (or CD45.1+).

☒ Tick this box to confirm that a figure exemplifying the gating strategy is provided in the Supplementary Information.
