# Supplementary material for: CD4+ T cell help creates memory CD8+ T cells with innate and help-independent recall capacities
Source: Nat Commun. 2019 Dec 4;10:5531. doi: 10.1038/s41467-019-13438-1 (PMC6892909; doi:10.1038/s41467-019-13438-1)
Supplement: Supplementary file 3 — Description of Additional Supplementary Files [file 41467_2019_13438_MOESM3_ESM.pdf]

Description of Additional Supplementary Files

File Name: Supplementary Data 1

Description: All 193 genes that were found to be differentially expressed between helped and helpless E7-specific T_{CM} cells at the steady-state, as described in Figure 3.

File Name: Supplementary Data 2

Description: All 2522 genes that were found to be differentially expressed between helped and helpless E7-specific T_{EM} cells at the steady-state, as described in Figure 3.

File Name: Supplementary Data 3

Description: All 1315 genes differentially expressed between helped and helpless effector CTLs in the secondary response, as described in Figure 5.

File Name: Supplementary Data 4

Description: All 391 genes differentially expressed between helped and helpless effector CTLs in both primary and secondary response, as described in Figure 5.

File Name: Supplementary Data 5

Description: All 4643 differentially modified regions (DMR) as designated by H3K4me3 marks between helped and helpless memory cells, as described in Figure 6.

File Name: Supplementary Data 6

Description: All 882 differentially modified regions (DMR) as designated by H3K27me3 marks between helped and helpless memory cells, as described in Figure 6.

File Name: Supplementary Data 7

Description: Summary of quality control metrics of ChIPseq experiment described in Figure 6. Peaks = number of peaks called in the sample, Reads = reads in the library, Dup% = number of duplicate reads, ReadL = read length, FragL = fragment length, RelCC = relative cross-coverage score, SSD = squared sum of deviations, RiP% = percent reads in peaks.

File Name: Supplementary Data 8

Description: Details of antibodies used for flow cytometric analysis.
